# Immunomodulatory potential of Mongolian medicine in sepsis: current evidence, challenges, and translational perspectives

**DOI:** 10.3389/fimmu.2026.1888250

**Published:** 2026-10-01

**Authors:** Shaohua Cui, Di Wu, Yongli Zhang, Lixia Geng

**Affiliations:** 1 Department of Critical Care Medicine, The First Affiliated Hospital of Baotou Medical College, Inner Mongolia University of Science and Technology, Baotou, China; 2 Graduate School, Dalian Medical University, Dalian, Liaoning, China; 3 Department of Critical Care Medicine, The First Affiliated Hospital of Dalian Medical University, Dalian, Liaoning, China

**Keywords:** gut microbiota, immune checkpoints, immune dysregulation, immunometabolism, immunomodulation, inflammasome, Mongolian medicine, sepsis

## Abstract

Sepsis is a heterogeneous, time-dependent syndrome in which inflammatory injury, immune suppression, endothelial dysfunction, metabolic disturbance, and barrier failure may coexist. This narrative review evaluates whether current pharmacological evidence provides a defensible basis for investigating Traditional Mongolian Medicine as an adjunctive source of immunomodulatory candidates in sepsis. A concise account of sepsis immunopathology is used as an interpretive framework, after which evidence from named Mongolian formulas, extracts, medicinal materials, and isolated constituents is mapped according to ethnomedical specificity, model directness, and study-design limitations. Direct sepsis-specific evidence is scarce. Daruqi particles, HHQG, Hohgardi-9, Nagab-9, Monflu, Batri-7, Qiqirigan-8, and selected extracts have mainly been studied in lipopolysaccharide-induced acute lung injury, organ-injury, enteritis, inflammatory, or metabolic models. These studies suggest effects on NF-κB/MAPK signaling, myeloid-cell infiltration, neutrophil extracellular traps, redox regulation, and lipid metabolism, but generally do not assess polymicrobial infection, pathogen clearance, survival, late immunosuppression, or human immune endotypes. Widely distributed compounds such as quercetin, berberine, geniposide, and gallate are therefore treated only as non-specific constituent-level evidence of pharmacological plausibility. We propose candidate-specific priorities for chemical standardization, rigorous sepsis modeling, pharmacokinetic and safety evaluation, and biomarker-guided translation. The current literature supports an ethnopharmacology-informed research agenda, not therapeutic efficacy in sepsis.

## 1 Introduction

Sepsis is life-threatening organ dysfunction caused by a dysregulated host response to infection (1). Despite advances in antimicrobial therapy, source control, and organ support, it remains a major cause of death, prolonged hospitalization, and long-term disability (1, 2). Its biology cannot be reduced to cytokine excess: inflammatory, immune, endothelial, coagulation, metabolic, and tissue-injury processes interact over time (3, 4).

This heterogeneity helps explain why broadly anti-inflammatory interventions have repeatedly failed. Hyperinflammation can coexist with monocyte deactivation, lymphopenia, impaired antigen presentation, and T-cell exhaustion, while infection source, pathogen burden, age, comorbidity, microbiome configuration, and treatment timing further modify the response (5–8). Host-directed therapy therefore requires attention to immune state and disease stage rather than indiscriminate suppression.

Natural products, dietary bioactives, and traditional medical systems are relevant to this discussion because multi-component exposures can affect several connected biological processes (9–12, 36). Traditional Chinese medicine and other natural-product approaches have been reviewed extensively in sepsis, whereas Mongolian medicine has received comparatively little critical attention in the international immunology literature (9–13).

Mongolian medicine includes named formulas, medicinal materials, extracts, and constituent compounds used historically for febrile, respiratory, gastrointestinal, and inflammatory disorders (13). Recent experiments report effects on innate inflammatory signaling, oxidative stress, tissue injury, and metabolic regulation (14–16). These observations justify candidate selection, but do not establish clinical or disease-specific efficacy.

The interpretive boundary used throughout this review is as follows: evidence from non-infectious or lipopolysaccharide-only models may support pathway plausibility, but it cannot by itself demonstrate benefit in sepsis. Therapeutic relevance requires preservation of pathogen clearance, improvement in clinically meaningful outcomes, and assessment across the hyperinflammatory and immunosuppressive phases of disease.

The review therefore asks which Mongolian medicine-specific preparations have been studied, what sepsis-relevant biological domains those studies address, and which evidentiary gaps prevent translation. Formula- and extract-level findings are considered separately from results for widely distributed natural compounds, which are not counted as validation of Mongolian medicine as a therapeutic system.

The aims are to provide a concise immunological framework, critically map the available Mongolian medicine-related evidence, define a transparent directness scale, and identify candidate-specific experimental priorities. The intended output is a narrative evidence map and research agenda rather than a systematic estimate of treatment effects.

### 1.1 Review scope and evidence identification

This narrative review was informed by structured searches of PubMed, Web of Science, Embase, China National Knowledge Infrastructure, SinoMed, and Wanfang Data from database inception to February 2026, supplemented by targeted reference checks during revision. English and Chinese terms combined Mongolian medicine, named formulas or medicinal materials, sepsis, septic shock, inflammation, immunomodulation, and related immune pathways.

The searches were used to identify mechanistically relevant literature for a narrative evidence map; they were not conducted under a prospectively registered systematic-review protocol. No claim is made that the resulting set is PRISMA-complete, and no retrospective screening counts are presented. Reports were categorized by intervention specificity (named formula, extract or medicinal material, or non-specific constituent), disease-model directness, and design/reporting limitations.

## 2 Sepsis immunopathology: concise interpretive framework

### 2.1 Coexisting immune injury and immune suppression

Pathogen-associated and damage-associated molecular patterns activate pattern-recognition receptors and downstream NF-κB, MAPK, interferon, complement, and inflammasome programs (3, 4, 17, 18). These responses are required for antimicrobial defense, but spatially or temporally dysregulated activation promotes cytokine release, leukocyte recruitment, vascular leak, coagulation abnormalities, and organ injury.

At the same time, many patients develop reduced monocyte HLA-DR expression, dendritic-cell loss, lymphocyte apoptosis, inhibitory-checkpoint upregulation, and impaired ex vivo cytokine responses (5, 6, 18, 19). Hyperinflammation and immune suppression should therefore be treated as overlapping, patient-specific states rather than fixed sequential phases.

### 2.2 Endothelial, thrombotic, metabolic, and barrier dysfunction

Neutrophil activation and extracellular traps, platelet and complement activation, endothelial injury, and immunothrombosis connect inflammation to microvascular obstruction and tissue damage (20, 22–25). Mitochondrial dysfunction and altered glucose, lipid, and lactate metabolism further reshape myeloid and lymphocyte function (38, 39).

The intestinal barrier and microbiota are additional components of this network. Dysbiosis, epithelial injury, microbial translocation, and altered microbial metabolites can amplify systemic inflammation and organ dysfunction (50–57). These processes provide relevant experimental domains, but none is a surrogate for survival, pathogen control, or recovery from sepsis.

### 2.3 Implications for evaluating traditional interventions

A candidate intervention should be judged against the dysfunction it is intended to modify. Suppression of TNF-α, IL-6, or NF-κB in an isolated model is informative only at the pathway level; sepsis-oriented studies must also measure microbial burden, immune-cell function, endothelial or organ injury, treatment timing, and survival.

This distinction is especially important for Mongolian medicine because most available studies address innate inflammatory signaling in non-sepsis settings. The evidence map below therefore separates pharmacological observations from disease-specific validation and identifies domains for which no Mongolian medicine-specific evidence currently exists.

### 2.4 Integrated framework

**Figure 1** summarizes the coexisting and time-varying components of septic immune dysregulation. It is used only as a framework for locating candidate mechanisms; it does not imply that all patients follow a uniform early-to-late sequence.

**Figure 1:**
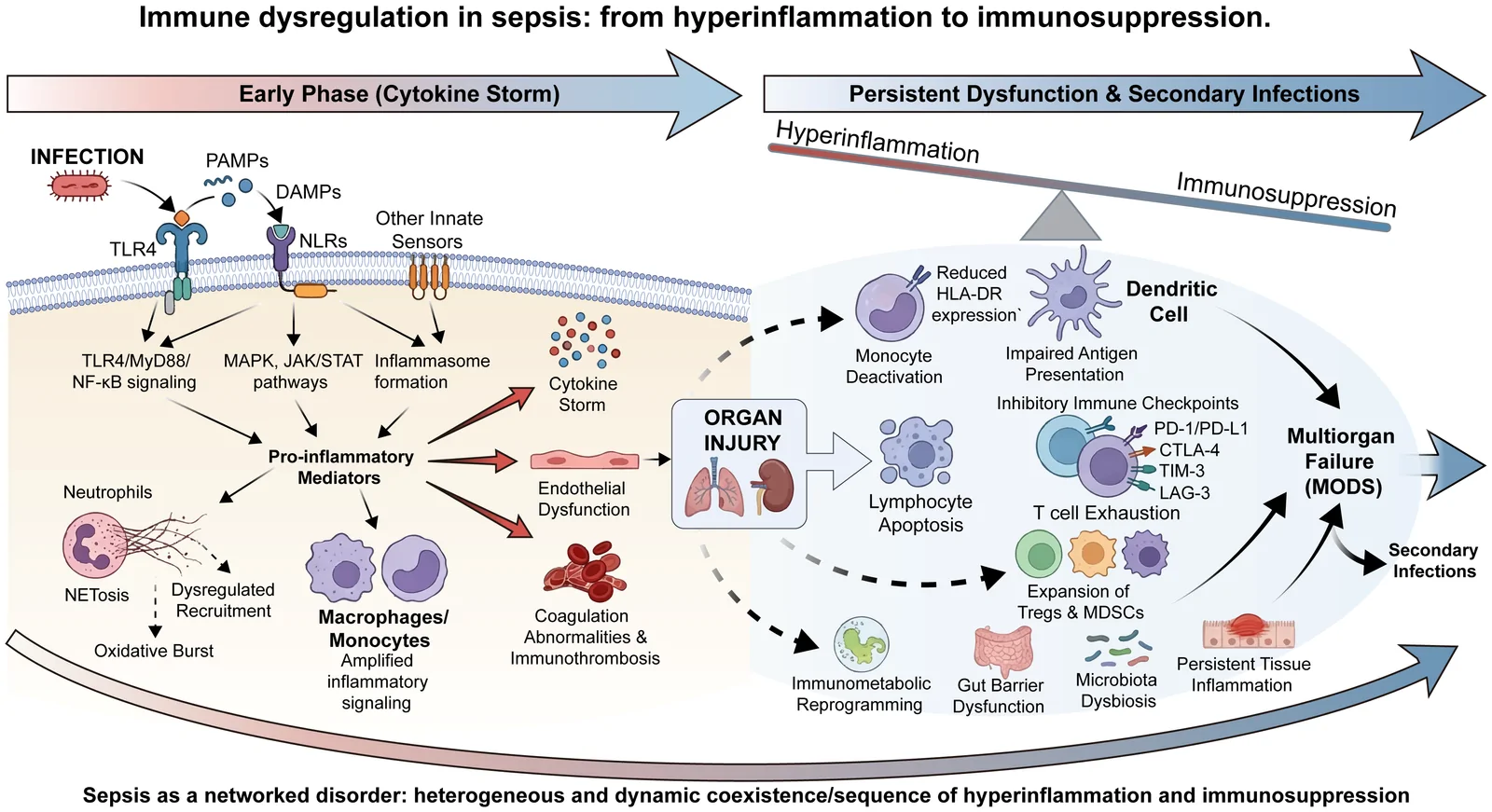
Immune dysregulation in sepsis. Infection triggers interacting inflammatory, immune-suppressive, endothelial, thrombotic, metabolic, and barrier responses. Hyperinflammation and immune suppression may coexist and vary across patients and time; the diagram should not be read as a mandatory linear progression. This framework is used to identify measurable domains for evaluating candidate interventions.

Within this framework, the strongest translational questions concern whether a defined preparation can improve a measurable immune or organ phenotype without compromising antimicrobial defense.

The pathways most relevant to the evidence map are summarized briefly below.

## 3 Evidence-assessment axes for candidate interventions

### 3.1 Innate inflammatory and inflammasome signaling

TLR4/MyD88/NF-κB and MAPK signaling, NLRP3 activation, and pyroptosis are prominent in experimental sepsis and organ injury (3, 17, 33–35). These pathways are frequently measured in natural-product studies, but pathway inhibition alone does not establish a net benefit during infection.

### 3.2 NETs, endothelial injury, and organ damage

Neutrophil extracellular trap formation is context-dependent (21), and excessive NETs in sepsis can promote endothelial damage, immunothrombosis, and lung injury (22–25, 37). Candidate studies should therefore pair NET and inflammatory readouts with pathogen clearance, vascular function, and organ-specific outcomes.

### 3.3 Adaptive immunity, immunometabolism, and the gut barrier

Lymphocyte apoptosis, PD-1/PD-L1 and related checkpoints, regulatory-cell expansion, metabolic exhaustion, and gut-barrier failure contribute to persistent immune dysfunction (18, 27–32, 38, 39, 50–57). For Mongolian medicine, these domains remain largely hypothesis-generating because direct formula-level studies are scarce.

## 4 Traditional Mongolian medicine: pharmacological basis and immunological relevance

Traditional Mongolian medicine is a northern Asian ethnomedical system that uses complex prescriptions and medicinal materials for disorders described in terms of fever, respiratory symptoms, gastrointestinal disturbance, inflammation, and systemic decline (13). Its preparations contain chemically diverse compounds, including flavonoids, phenolics, alkaloids, terpenoids, and polysaccharides (13–16).

Three features justify a focused research agenda: the ethnomedical use of named formulas, preliminary pharmacology in inflammatory and organ-injury models, and the possibility of testing standardized multi-component preparations against defined sepsis-relevant endpoints. These features support candidate prioritization, not an assumption of therapeutic superiority or clinical efficacy.

Accordingly, the review evaluates each preparation according to its ethnomedical specificity, experimental model, observed biological effect, and distance from clinically relevant sepsis.

### 4.1 Conceptual background and its limits in modern interpretation

Within Mongolian medical theory, Heyi, Xila, and Badagan are traditional explanatory categories used to describe balance, constitution, and disease. Historical accounts of febrile or systemic illness help explain why certain formulas were selected for symptoms involving fever, respiratory distress, gastrointestinal disturbance, or general deterioration.

These categories are not mapped onto cytokines, immune endotypes, metabolism, or stages of sepsis. No biological homology is proposed. They are included exclusively as ethnomedical rationale; all mechanistic interpretation in this review is based on contemporary experimental pharmacology and sepsis immunology.

### 4.2 What qualifies as Mongolian medicine-related evidence?

Evidence was organized along two independent dimensions: intervention specificity and disease-model directness. The categories are intended to prevent a pharmacological observation from being mistaken for validation of a therapeutic system.

Named-formula evidence has the greatest Mongolian medicine specificity. Extract- or medicinal-material-level evidence may identify useful activity, but it does not establish the behavior of a complete formula and remains sensitive to source, processing, and extraction methods.

Isolated compounds that are widely distributed across plants or medical traditions are classified separately as non-specific constituent-level evidence. Quercetin, berberine, geniposide, gallate, and similar compounds may support a molecular hypothesis, but they are not counted as Mongolian medicine-specific evidence.

Directness is not synonymous with study quality. The A/B/C labels used below are a custom scale describing proximity to sepsis, whereas design features such as randomization, blinding, sample-size justification, dose rationale, and outcome selection are reported as separate limitations.

### 4.3 Current pharmacological signals

The most reproducible signals concern innate inflammatory pathways, particularly NF-κB- and MAPK-associated signaling, cytokine production, myeloid-cell infiltration, oxidative stress, and acute lung injury (16, 40–47). Nagab-9 additionally reduced neutrophil infiltration and NET-related markers in an LPS-induced lung-injury model (44), while Qiqirigan-8 affected lipid metabolism and inflammatory parameters in obesity (42).

These models remain indirect. Most do not include live infection, polymicrobial sepsis, delayed treatment, pathogen burden, survival, late immune suppression, or patient-derived immune phenotyping. Several also rely on pretreatment designs or network-pharmacology predictions that require independent experimental validation.

### 4.4 Representative evidence and critical interpretation

**Table 1** maps named Mongolian formulas and extracts separately from non-specific constituents. It records the model, principal observation, custom directness category, and the main design or reporting limitation relevant to sepsis interpretation.

**Table 1:** Evidence map of Mongolian medicine-specific preparations and non-specific constituent-level pharmacological signals.

Intervention and specificity	Model	Main observation	Directness	Main design/reporting limitation	Ref
Daruqi particles (named formula)	Inflammation-related experimental systems	Anti-inflammatory pathway modulation	B	No infection-based sepsis validation; pathogen clearance and survival not assessed	(16)
HHQG/Guri Gumu-13 (named formula)	CCl4-induced acute liver injury	Reduced monocyte/macrophage infiltration and p38/JNK-associated signaling	B	Chemical liver injury rather than infection; no septic pharmacokinetics or survival outcomes	(40)
Hohgardi-9 (named formula)	LPS-induced ALI in rats	Reduced lung injury, edema, and inflammatory-gene expression	B	Pretreatment endotoxin model; small endpoint groups; no pathogen burden, survival, or clear blinding report	(43)
Nagab-9 (named formula)	LPS-induced ALI in mice and epithelial-cell injury	Reduced neutrophil infiltration, NET markers, and SRC/ERK1/2/STAT3 signaling	B	Network pharmacology plus endotoxin validation; no live infection, pathogen clearance, or late immune outcomes	(44)
Monflu/Lider-7-derived granule (named formula)	LPS-induced ALI in Wistar rats	Reduced cytokines and histological lung injury after pretreatment	B	Reported randomization, but animal totals and group allocation are internally inconsistent; no blinding, sample-size rationale, infection, or survival outcome	(45)
Batri-7 (named formula)	Salmonella enteritis and LPS-stimulated macrophages	Reduced intestinal inflammation and macrophage NO production	B	Localized enteritis/*in vitro* evidence; no systemic sepsis, bacteremia, organ failure, or survival endpoint	(46)
Qiqirigan-8 (named formula)	Obesity and metabolic inflammation	Altered lipid metabolism and inflammatory parameters	B	Metabolic disease model; no sepsis-specific immune or pathogen outcomes	(42)
Rhaponticum uniflorum inflorescence extract	LPS-mediated ALI	Reduced inflammatory injury through Nrf2/HO-1-associated signaling	B	Extract-level pretreatment/endotoxin evidence; no infection, pathogen clearance, or adaptive-immune assessment	(47)
Xanthium mongolicum fractions	Rheumatoid arthritis	Altered M1 macrophage polarization through NF-κB/MAPK signaling	B	Autoimmune model; no infection-based sepsis validation	(41)
Non-specific constituent-level evidence: pharmacological plausibility only; not counted as Mongolian medicine-specific validation					
Geniposide, gallate, and berberine mixture	LPS-stimulated RAW264.7 cells	Combined anti-inflammatory activity	C	*In vitro* cell-line study; compounds are not tradition-specific and were not tested in sepsis	(48)
Sophora alopecuroides constituents	LPS-stimulated RAW264.7 cells	Inhibited nitric oxide production	C	Isolated constituents *in vitro*; no formula-level or sepsis-specific validation	(49)
Quercetin	Microglial neuroinflammation	Modulated NLRP3-mitophagy signaling	C	Widely distributed compound; non-sepsis and non-formula evidence	(15)

No included preparation currently meets Level A criteria for direct sepsis-specific evidence. The table should therefore be read as a candidate and gap map, not as a hierarchy of proven therapies or a formal GRADE, OCEBM, or risk-of-bias assessment.

### 4.5 Scope of the pathway-oriented synthesis

Subsequent sections concentrate on domains for which at least a plausible candidate link can be specified. Adaptive immunity, checkpoint biology, immunometabolism, and microbiota-mediated effects are explicitly identified as hypothesis-generating where formula-level evidence is absent.

Custom directness scale: Level A, evidence from infection-based sepsis models, septic patient-derived systems, or clinical studies with sepsis-relevant outcomes; Level B, indirect evidence from endotoxin, non-sepsis inflammatory, organ-injury, or metabolic models; Level C, exploratory non-specific constituent or *in vitro* evidence without sepsis validation. These labels describe directness, not methodological quality. Design/reporting limitations are listed separately. Abbreviations: ALI, acute lung injury; CLP, cecal ligation and puncture; NETs, neutrophil extracellular traps.

**Figure 2** places these observations within a pathway framework while preserving the same evidence boundary.

**Figure 2:**
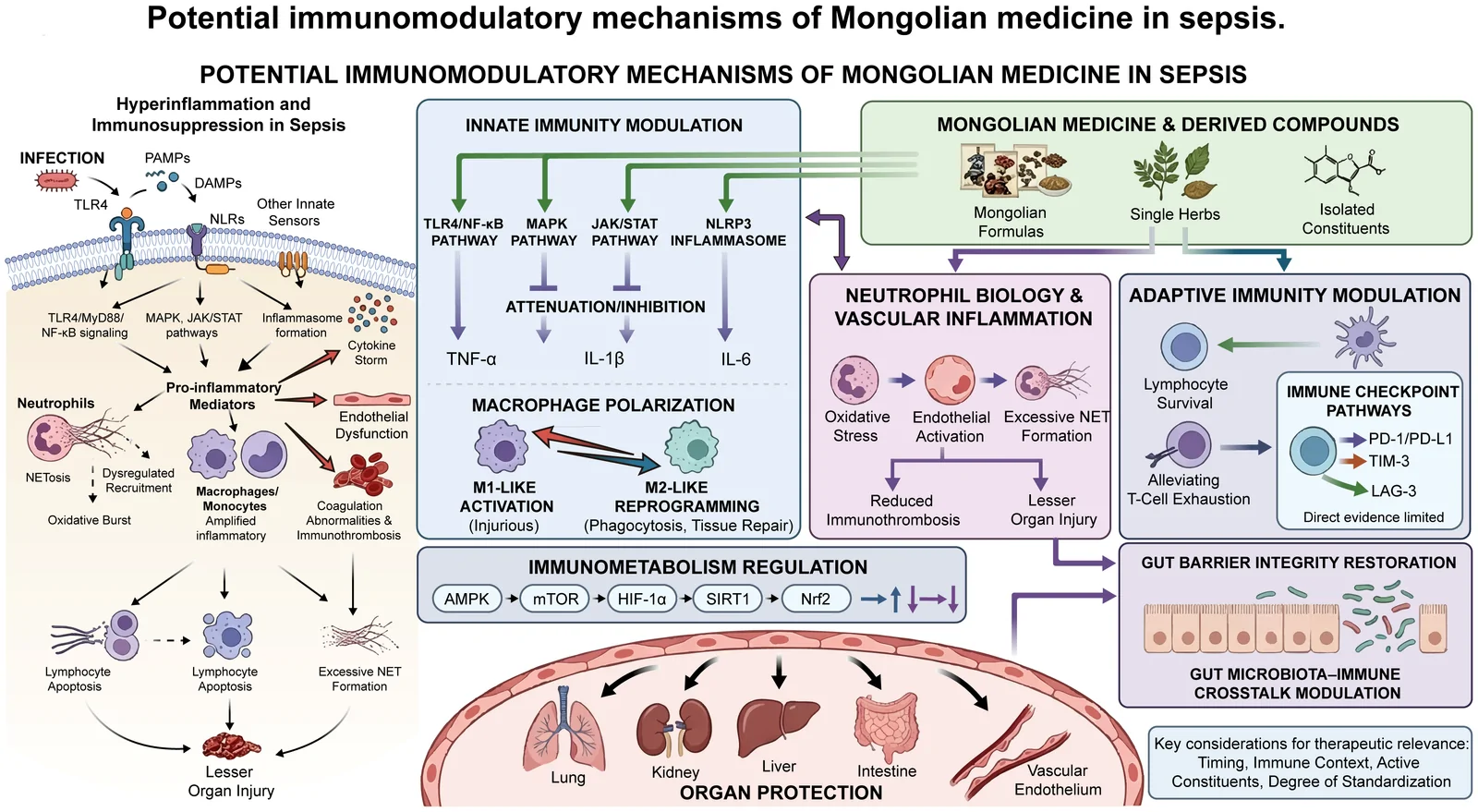
Potential immunomodulatory mechanisms of Mongolian medicine in sepsis. Solid lines indicate pathways for which some pharmacological observations have been reported for a named Mongolian formula, extract, or included constituent. Most such observations arise from LPS-induced acute lung injury, non-sepsis inflammatory or organ-injury models, or *in vitro* studies; a solid line therefore denotes indirect pharmacological support, not sepsis-specific validation. Dotted lines indicate links that remain hypothesis-generating. No line style should be interpreted as evidence of clinical efficacy.

The figure is intended to guide testable experiments. It does not establish that a multi-component preparation restores systemic immune homeostasis or that effects observed in one tissue will translate across sepsis endotypes.

## 5 Potential effects of Mongolian medicine on innate immunity in sepsis

Innate immunity is the best-supported entry point because the available formula and extract studies predominantly measure macrophage, neutrophil, cytokine, oxidative, or tissue-injury responses. The evidence remains Level B unless infection-based sepsis outcomes are assessed.

### 5.1 Monocyte/macrophage-centered inflammatory regulation

Monocytes and macrophages contribute to both antimicrobial defense and inflammatory tissue injury through NF-κB-, MAPK-, inflammasome-, and metabolic programs (3, 17, 20, 35). Interpretation therefore requires more than a reduction in circulating cytokines.

Daruqi particles showed anti-inflammatory activity (16); HHQG reduced monocyte/macrophage infiltration in CCl4-induced liver injury (40); and Xanthium mongolicum fractions altered M1 macrophage polarization in rheumatoid arthritis (41). These results identify myeloid pathways for follow-up but do not demonstrate efficacy in infection-driven sepsis.

### 5.2 Neutrophil dysregulation and immunothrombosis

Neutrophils control pathogens through phagocytosis, degranulation, reactive oxygen species, and NET formation, whereas excessive NETs promote endothelial injury and immunothrombosis (22–24). Both antimicrobial and tissue-injury outcomes must therefore be measured.

Nagab-9 reduced neutrophil infiltration and the NET-related markers Ly6G and citrullinated histone H3 in LPS-induced acute lung injury, with SRC/ERK1/2/STAT3 signaling implicated (44). This is a formula-specific pharmacological signal, but the non-infectious endotoxin model did not establish effects on microbial clearance, survival, or late immune suppression.

### 5.3 Dendritic cell dysfunction and innate-adaptive immune coordination

Dendritic-cell loss and impaired antigen presentation contribute to sepsis-associated immune paralysis (5, 26).

No study in the mapped Mongolian medicine literature directly tested dendritic-cell number, antigen presentation, or T-cell priming. Claims of restored innate-adaptive coordination would therefore be premature.

### 5.4 Inflammasome activation and pyroptosis

NLRP3 activation and pyroptosis contribute to sepsis-associated tissue injury (33, 34). No mapped named formula has yet been tested for pyroptosis in infection-based sepsis.

Quercetin modulated the NLRP3-mitophagy axis in a microglial neuroinflammation model (15). Because quercetin is widely distributed and the model was unrelated to sepsis, this finding is retained only in the non-specific constituent category.

### 5.5 Endothelial injury, tissue infiltration, and organ dysfunction

Endothelial activation and vascular-barrier failure are central to sepsis (20, 25, 35), but the mapped Mongolian medicine studies do not directly assess endothelial function, microvascular perfusion, or coagulation in infection-based models.

### 5.6 Immunometabolism and the intestinal innate immune interface

Metabolic state and the intestinal barrier shape innate immune behavior (38, 39, 50–57). Qiqirigan-8 altered lipid metabolism and inflammation in obesity (42), while Batri-7 was studied in Salmonella enteritis and LPS-stimulated macrophages (46).

These preparations provide candidate-specific starting points for immunometabolic and gut-barrier experiments, but neither study demonstrates correction of septic immunometabolism or prevention of systemic microbial translocation.

## 6 Hypothesis-generating domains in adaptive immunity

### 6.1 Lymphocyte survival and checkpoint biology

Lymphocyte apoptosis and increased PD-1/PD-L1, TIM-3, LAG-3, and CTLA-4 signaling contribute to sepsis-associated immune dysfunction (27–31). These pathways are clinically relevant, but they have not been directly examined for the mapped Mongolian formulas.

A defensible study would combine lymphocyte survival, checkpoint expression, proliferation, cytokine production, microbial clearance, and survival across defined time points. Until such data exist, adaptive immune restoration should be described as a research question rather than an expected effect.

### 6.2 Regulatory-cell balance and adaptive recovery

Treg, MDSC, and Th17/Treg dynamics are time-dependent and can be either protective or harmful (18, 32). Broad increases or decreases in these populations cannot be assumed to be beneficial.

No formula-level study in the mapped literature measured these populations or their suppressive function. Candidate studies should therefore begin with immune phenotyping and functional assays rather than pathway prediction alone.

## 7 Immunometabolism: candidate-specific questions

Sepsis alters glycolysis, mitochondrial respiration, lactate handling, and nutrient sensing in myeloid and lymphoid cells (38, 39). Qiqirigan-8 is the most directly relevant named candidate because it affected lipid metabolism and inflammatory parameters in an obesity model (42), although this model does not reproduce septic metabolic failure.

Follow-up should test Qiqirigan-8 and chemically defined fractions in infection-based sepsis using metabolic flux, mitochondrial function, targeted metabolomics, immune-cell phenotyping, pathogen burden, and survival. AMPK, mTOR, HIF-1α, SIRT1, and Nrf2 should be treated as testable targets, not established mechanisms.

## 8 Gut microbiota-immune axis and mucosal barrier

### 8.1 Relevance and current evidence

Intestinal injury, dysbiosis, altered microbial metabolites, and translocation can amplify systemic inflammation and organ dysfunction in sepsis (50–57). Despite the oral administration of many Mongolian preparations, no mapped study directly demonstrates microbiota-dependent benefit in sepsis.

### 8.2 Candidate-specific research

Batri-7 is a logical candidate for gut-origin infection models because it has been studied in Salmonella enteritis (46). Qiqirigan-8 may be considered for metabolic-endotype studies (42). Experiments should distinguish direct anti-inflammatory effects from microbiota-mediated effects and include barrier permeability, viable bacterial translocation, microbial metabolites, immune phenotypes, and survival.

## 9 Organ-specific immune injury and possible intervention points

### 9.1 Sepsis-associated acute lung injury

Lung-directed evidence is the largest formula-specific cluster. Hohgardi-9, Nagab-9, Monflu, and Rhaponticum uniflorum extract reduced inflammatory or histological injury in LPS-induced ALI models (43–45, 47). These were endotoxin or pretreatment studies rather than infection-based pneumonia or polymicrobial sepsis, and they did not establish pathogen clearance or survival benefit (58).

### 9.2 Sepsis-associated acute kidney injury

Sepsis-associated acute kidney injury involves inflammatory, endothelial, mitochondrial, and pyroptotic mechanisms (34, 59). No mapped Mongolian formula has been directly evaluated in this setting; kidney-directed claims should remain prospective.

### 9.3 Liver injury and immunometabolic disruption

HHQG reduced monocyte/macrophage infiltration and p38/JNK-associated signaling in CCl4-induced acute liver injury (40). This supports a liver-injury candidate pathway, but chemical injury does not reproduce infection, septic pharmacokinetics, or systemic immune dysfunction.

### 9.4 Intestinal injury and distant organ amplification

Batri-7 and Qiqirigan-8 provide indirect starting points for gut and metabolic studies (42, 46). Their relevance should be tested in gut-origin sepsis with barrier, bacterial-translocation, immune, and distant-organ endpoints (50–57).

### 9.5 Endothelial dysfunction and cardiovascular injury

No mapped Mongolian medicine study directly assessed endothelial permeability, microvascular perfusion, or immunothrombosis in sepsis (60). This remains an evidence gap rather than a supported mechanism.

## 10 Limitations and a Mongolian medicine-specific research agenda

**Table 2** consolidates the previous limitations and roadmap tables. It links each evidence gap to named Mongolian preparations, required experiments, and progression criteria.

**Table 2:** Current limitations and candidate-specific research priorities for Mongolian medicine in sepsis.

Evidence gap	Why it matters	Mongolian medicine-specific priority	Candidate preparation(s)	Progression criterion
Disease-model directness	Endotoxin and non-sepsis injury do not reproduce infection, pathogen burden, or immune trajectories	Test delayed treatment in pneumonia-derived sepsis, CLP, or fecal peritonitis; measure pathogen burden, organ injury, immune state, and survival	Nagab-9; Hohgardi-9; Monflu; Rhaponticum extract; Daruqi	Reproducible benefit without impaired pathogen clearance or late immune suppression
Chemical and batch standardization	Variable materials and processing undermine reproducibility	Define authenticated ingredients, manufacturing process, quantitative markers, fingerprints, stability, and batch-acceptance limits	Daruqi; HHQG; Nagab-9; Hohgardi-9; Monflu/Lider-7	Comparable exposure and biological activity across independent batches
Experimental rigor	Incomplete reporting inflates uncertainty	Prespecify randomization, concealment, blinding, sample-size calculation, exclusions, dose rationale, and adverse events; follow ARRIVE	All animal candidates	Independent replication with transparent reporting and consistent primary outcomes
Myeloid and NET mechanisms	Reduced cytokines alone do not establish preserved host defense	Pair NET/myeloid phenotyping with bacterial killing, vascular injury, and survival	Nagab-9; HHQG; Xanthium fractions	Mechanistic effect persists in infection-based models without worse bacterial burden
Gut barrier and immunometabolism	Oral formulas may act through barrier or metabolic pathways, but direct evidence is absent	Use gut-origin sepsis, permeability, viable translocation, microbial metabolites, immune-cell metabolism, and organ cross-talk	Batri-7; Qiqirigan-8	Barrier or metabolic benefit is causally linked to improved infection-based outcomes
Pharmacokinetics and safety	Sepsis changes absorption, distribution, metabolism, excretion, and drug interactions	Measure exposure, tissue distribution, organ toxicity, and interactions with antimicrobials and critical-care drugs under septic conditions	Prioritized standardized formulas or fractions	Defined exposure-safety window and no clinically relevant interaction signal
Human translation	Broad efficacy trials would be premature and confounded by sepsis heterogeneity	Begin with human pharmacology/safety, then biomarker-enriched adjunctive trials with prespecified immune and clinical endpoints	Only candidates meeting the preceding criteria	Standardized product, mechanistic target engagement, acceptable safety, and credible responder definition

### 10.1 Evidence directness and experimental rigor

The current evidence is dominated by endotoxin, non-sepsis organ-injury, inflammatory, metabolic, and *in vitro* models. Future animal studies should prespecify randomization, allocation concealment, blinding, sample-size justification, dose rationale, exclusions, and adverse-event reporting in line with ARRIVE principles. A future systematic synthesis should use a protocol-based risk-of-bias tool such as SYRCLE rather than retrospectively presenting the present narrative map as a formal grade of certainty.

### 10.2 Formula-specific priorities

Nagab-9, Hohgardi-9, Monflu, and Rhaponticum uniflorum extract should first be tested in pneumonia-derived or polymicrobial sepsis because their current evidence is lung- and endotoxin-centered (43–45, 47). Daruqi and HHQG require better-defined models and chemical characterization (16, 40). Batri-7 is suited to gut-origin infection and barrier studies (46), whereas Qiqirigan-8 is better positioned for immunometabolic endotypes (42).

### 10.3 Standardization, pharmacology, and safety

Each candidate should have authenticated source materials, manufacturing details, quantitative chemical markers, batch-acceptance criteria, and stability data. Pharmacokinetics, tissue exposure, organ toxicity, and herb-drug interactions should be studied under septic conditions before efficacy claims are advanced.

### 10.4 Sequencing clinical translation

Clinical testing should not begin with broad phase II/III efficacy claims. A candidate should progress only after reproducible infection-based benefit without impaired pathogen clearance, followed by human pharmacology and safety assessment. Early trials should evaluate a standardized adjunct, not a stand-alone anti-sepsis treatment, in a biomarker-defined population with prespecified immune and clinical endpoints.

## 11 Conclusion

Mongolian medicine offers several formula- and extract-level candidates for sepsis research, particularly in lung injury, myeloid inflammation, gut-origin infection, and immunometabolism. The strongest signals involve Nagab-9, Hohgardi-9, Monflu, HHQG, Batri-7, Qiqirigan-8, Daruqi, and Rhaponticum uniflorum extract, but all remain indirect with respect to infection-based sepsis.

The appropriate conclusion is therefore limited: current studies identify pharmacological leads and testable mechanisms, not therapeutic efficacy. Progress depends on candidate-specific chemical standardization, rigorous infection-based models, pathogen and survival outcomes, pharmacokinetic and safety data, and biomarker-guided human studies. Non-specific constituents should remain supporting mechanistic observations rather than evidence for Mongolian medicine as a distinct therapeutic system.

## Data Availability

The original contributions presented in this review are included in the article and its cited sources. Further inquiries can be directed to the corresponding authors.
